# Fracture resistance and bonding performance after antioxidants pre-treatment in non-vital and bleached teeth

**DOI:** 10.1590/0103-6440202305553

**Published:** 2023-10-27

**Authors:** Aryvelto Miranda Silva, Joissi Ferrari Zaniboni, Cristiane de Melo Alencar, Edson Alves de Campos, Andrea Abi Rached Dantas, Milton Carlos Kuga

**Affiliations:** 1 Department of Dentistry, Federal University of Juiz de Fora, Campus GV (UFJF-GV), Governador Valadares, MG, Brazil; 2 Department of Restorative Dentistry, Araraquara Dental School, São Paulo State University (UNESP), Araraquara, SP, Brazil.; 3 Graduate Program in Dentistry, University Center of the State of Pará (CESUPA), Belém, PA, Brazil.

**Keywords:** Tooth bleaching, antioxidants, fracture resistance, bond strength

## Abstract

This study aimed to evaluate the effect of antioxidant solutions on fracture strength and bonding performance in non-vital and bleached (38% hydrogen peroxide) teeth. One hundred and eighty dentin specimens were obtained, 60 for each test: fracture strength, hybrid layer thickness, and bond strength. The groups (n=10) were randomly composed according to post-bleaching protocol: REST - restoration, without bleaching; BL - bleaching + restoration; SA - bleaching, 10% sodium ascorbate solution, and restoration; AT - bleaching, 10% α-tocopherol solution, and restoration; CRAN - bleaching, 5% cranberry solution, and restoration; CAP - bleaching, 0.0025% capsaicin solution, and restoration. Data were analyzed with ANOVA, Kruskal-Wallis, Dunn, and Qui-Square tests (α=0.05). The highest fracture strength values were observed in REST (1508.96 ±148.15 N), without significant difference for the bleached groups (p>0.05), regardless of the antioxidant use. The hybrid layer thickness in the group that was not subjected to bleaching (REST) was significantly higher than in any other group. The bond strength in the bleached and antioxidants-treated groups (SA, AT, CRAN, CAP) has no differences with the bleached group without antioxidants (BL). Adhesive failures were predominant in the groups that did not receive the antioxidant application. In conclusion, the evaluated antioxidants did not show an effect on the fracture strength, hybrid layer thickness, or bond strength of dentin bleached after endodontic treatment. The application of 10% sodium ascorbate, 10% alpha-tocopherol, 5% cranberry, or 0.0025% capsaicin solutions is not an effective step and should not be considered for the restorative protocols after non-vital bleaching.

## Resumo

Este estudo teve como objetivo avaliar o efeito de soluções antioxidantes na resistência à fratura e resistência de união em dentes tratados endodonticamente e clareados (38% de peróxido de hidrogênio). Cento e oitenta espécimes de dentina foram obtidos, 60 para cada teste: resistência à fratura, espessura da camada híbrida e resistência de união. Os grupos (n=10) foram compostos aleatoriamente de acordo com o protocolo pós-clareamento: REST - restauração, sem clareamento; BL - clareamento + restauração; SA - clareamento, solução de ascorbato de sódio a 10% e restauração; AT - clareamento, solução de α-tocoferol a 10% e restauração; CRAN - clareamento, solução de cranberry a 5% e restauração; CAP - clareamento, solução de capsaicina 0,0025% e restauração. Os dados foram analisados ​​com os testes ANOVA, Kruskal-Wallis, Dunn e Qui-Quadrado (α=0,05). Os maiores valores de resistência à fratura foram observados em REST (1508,96 ±148,15 N), sem diferença significativa para os grupos clareados (p>0,05), independente do uso de antioxidantes. A espessura da camada híbrida no grupo que não foi submetido ao clareamento (REST) foi significativamente maior do que em qualquer outro grupo. A resistência de união nos grupos clareado e tratado com antioxidantes (SA, AT, CRAN, CAP) não apresentou diferenças com o grupo branqueado sem antioxidantes (BL). As falhas adesivas foram predominantes nos grupos que não receberam a aplicação do antioxidante. Em conclusão, os antioxidantes avaliados não mostraram efeito sobre a resistência à fratura, espessura da camada híbrida ou resistência de união à dentina clareada após tratamento endodôntico. A aplicação de soluções de ascorbato de sódio 10%, alfa-tocoferol 10%, cranberry 5% ou capsaicina 0,0025% não é uma etapa eficaz e não deve ser considerada para os protocolos restauradores após clareamento não vital.

## Introduction

Tooth bleaching in non-vital teeth carried out with hydrogen peroxide in variable concentrations (5 - 40%) has been proposed as a treatment to bleaching and improving the appearance of the tooth after endodontic treatment ^(^ ^1^. Hydrogen peroxide has a low molecular weight, high diffusion, and releases free radicals inside the dentinal tubules ^2^. The unpaired electrons present in free radicals are highly unstable, reactive, energetic, and rapidly react with oxygen ^3^. This reaction gives rise to reactive oxygen radical species (ROS) ^3^. Singlet oxygen, hydroxyl, and perhydroxyl ions are the ROS usually observed in oxidation reactions during bleaching ^4^.

ROS can combine with hydroxyapatite, producing apatite peroxide, causing phosphate/calcium degradation ^5^, thus reducing the fracture resistance of the tooth crown ^6^. In addition, ROS may affect adhesive bonding to the bleached dental structure ^7^. The residual oxygen ions tend to remain inside the dentinal tubules for a certain period, negatively affecting monomer infiltration into the etched dentin, and hampering a suitable hybrid layer formation ^8^ ^,^ ^9^. Furthermore, ROS can inhibit adhesives monomers polymerization or producing polymers with poor mechanical properties ^5^. ROS slowly dissipate over time in dental structures; thus, a recommended clinical practice is to postpone the bonding procedure, with a waiting period of 24 h to 4 weeks after bleaching, in order to avoid adhesive failures, since the reduction in bond strength to bleached enamel/dentin is temporary ^10^ ^,^ ^11^.

Recently, antioxidant agents have been studied in order to neutralize the negative effects of ROS, and reverse the bond strength, and damages to the dental structure when applied immediately after bleaching and before the final restoration ^7^ ^,^ ^10^. Antioxidants donate electrons to free radicals, ending the electron-stealing reaction, and increasing the reduction potential of the enamel/dentin surface ^12^ ^,^ ^13^. Thus, antioxidants may have a positive effect on fracture resistance, increasing bond strength, and adhesive polymerization, favoring restoration longevity ^9^. The use of antioxidants can be interesting in cases where the restorative procedure needs to be performed immediately after tooth bleaching.

The most common antioxidants are ascorbic acid, and its derivatives, such as sodium ascorbate ^14^ ^,^ ^15^. However, ascorbic acid has not been recommended after bleaching, as it may cause a potential double etching effect on teeth ^16^. Moreover, is unsuitable for clinical applications due to its low pH (1.8) ^15^. However, sodium ascorbate has a higher pH (7.4) and similar antioxidant properties ^17^. One of the components of vitamin E, Alpha-tocopherol is another antioxidant that has been evaluated ^7^ ^,^ ^18^.

In addition, naturally, occurring antioxidants have also been studied, such as pine bark, cranberry, grape seed extract, lemon tree bark, and hazelnut tree leaves ^10^. These natural antioxidants contain oligomeric proanthocyanidin complexes (OPCs) presenting a free radical scavenging ability that is 50-times more potent than sodium ascorbate and 20-times more than Vit E ^19^. Cranberries are rich sources of phytochemicals, having anti-cancer, anti-mutagenic, anti-inflammatory, anti-viral properties, and anti-bacterial agents, which prevents urinary tract infections. In addition, they present antioxidant properties ^20^. Cranberry has minimal positive effects on reversal immediate bond strength on bleached enamel ^13^. However, no studies have evaluated cranberry extract solution effects on bonding performance to bleached dentin.

This study has proposed another antioxidant, capsaicin (8-methyl-N-vanillyl-trans-6-nonenamide). Capsaicinoids are alkaloids in red pepper (Capsicum annuum) that present pharmacological properties, such as analgesia, anticancer, anti-inflammatory, anti-obesity, and antioxidant activity ^20^. In addition to a gap in the effectiveness of this specific antioxidant, the literature remains uncertain about reversing bond strength on bleached dental substrates ^7^ ^,^ ^10^.

Therefore, this study evaluated the hypothesis that post-bleaching treatment with sodium ascorbate, alpha-tocopherol, cranberry, or capsaicin solutions has no effect on fracture resistance, hybrid layer formation, and immediate bond strength to dentin in endodontically treated teeth.

## Materials and Methods

### Sample size calculation

Data obtained from a pilot study were considered for the sample size calculation. The calculation was carried out using the G Power software ^21^, considering the Analysis of Variance test (ANOVA - one way), effect size [0.36825 (fracture resistance), 0.42665 (hybrid layer) and 0.39771 (bond strength)], alpha = 0.05 and power of 80%. The calculations performed indicated that between 7 and 9 samples/group were minimally necessary. Considering this and other similar studies in the area, the size of 10 samples per group was defined.

### Sample preparation

The teeth with straight root, fully formed root apice, single root canal, without calcification or resorption, radiographically confirmed, and uniform length of 21mm (± 1mm) were considered for inclusion. The study included one hundred and eighty bovine incisors stored in thymol solution (0.1%; pH 7.0) in a humid environment (4ºC) for 7 days to disinfection and control of bacterial growth previously endodontic treatment was performed.

The teeth were subjected to endodontic treatment. The root lengths were standardized at 16 mm. Initially, access cavity was explored using a size 15 K-file (Dentsply Maillefer, Ballaigues, Switzerland) and enlarged up to a F3 instrument (ProTaper Universal; Dentsply Maillefer, Ballaigues, Switzerland). The working length was established 1 mm shorter than the standardized root length. All root canals were irrigated with distilled water during the cleaning and shaping preparation ^22^. After treatment, provisional restoration (IRM; Dentsply Ind. Com. Ltda., Petrópolis, Brazil) of cavity access was performed and the teeth were stored immersed in artificial saliva until the experiments were carried out.

### Experimental Groups

Six groups (n=10/group/experiment) were composed according to bleaching, post-bleaching, and restorative protocols, as can be seen in Table 1.

**Table 1:** Experimental groups composition according to bleaching, post-bleaching, and restorative protocols

Groups	Bleaching	Post-bleaching	Restoration
REST	-	-	+
BL	+	-	+
SA	+	+	+
AT	+	+	+
CRAN	+	+	+
CAP	+	+	+

In all experimental groups, restorations were performed. These were carried out according to the sequence: etching on the pulp chamber (30 s), adhesive system (Scotchbond Multi-Purpose; 3M ESPE, St. Paul, USA) application following manufacturer's recommendations, and pulp chamber restoration with composite resin (Filtek Z-250; 3M, St. Paul, USA) using light-activated increments of 2 mm (LED Bluephase; Ivoclar Vivadent, Schan, Liechtenstein, AL; 1.200mW/cm2; 40 s/increment).

The bleaching (BL, SA, AT, CRAN, and CAP groups) was carried out with 38% hydrogen peroxide (Opalescence Xtra Boost; Ultradent Products Inc., South Jordan, USA). The bleaching gel was applied to the external and internal surfaces of the tooth crown according to the manufacturer’s recommendations. In sequence, bleaching gel was removed with aspiration cannula, and the bleached surfaces were rinsed with water.

In the SA (10% sodium ascorbate solution), AT (10% alpha-tocopherol solution), CRAN (5% cranberry solution), and CAP (0,0025% capsaicin solution) groups the specimens received an antioxidants application after the bleaching. The antioxidants solutions were applied to the buccal surface and the pulp chamber for 15 and 10 min, respectively. In sequence, rinse was carried out with water for 15 s, and the restoration was performed immediately after as previously described.

### Fracture resistance and fracture pattern evaluation

The specimens from each group were individually placed in plastic matrices (16.5 mm diameter × 20.0 mm length) and filled with polyester resin (Maxi Rubber, São Paulo, Brazil) up to 2 mm below the cemento-enamel junction (CEJ). These root-matrix sets were stored for 24 hours to ensure complete resin polymerization ^23^.

The fracture strength test was conducted with an electromechanical testing machine (EMIC DL 2000; São José dos Pinhais, Brazil), with 5 kN load at 0.5 mm/min. An axial load was applied with an angle of 135º in relation to their long axis until the fracture occurs. This angulation simulates dental intercuspation ^24^. The force values applied before the fracture were recorded.

The fracture limit in relation to the CEJ was used as a parameter for the classification of the fracture pattern. Thus, the fractures that stopped more or less than 1 mm coronal to the CEJ were classified as favorable or unfavorable, respectively ^25^.

### Hybrid layer evaluation

Another 60 of the bovine incisors endodontically treated were allocated in the experimental groups (n=10) and received the treatments described earlier.

24 hours after completion of treatments, longitudinal sections in the middle third of the tooth crowns were performed. These cuts were performed in a cutting machine under intense cooling to avoid any damage in the hybrid layer formation. The specimens obtained (10 mm length ×5 mm width) were polished with a sequence of the #600 and #1200 water sandpapers (Norton, Lorena, SP, BR), distilled water rinse, and finishing using aluminum oxide paste (30 μm granulation; Arotec, São Paulo, SP, BR) and felt disk. In sequence, the specimens were rinsed in an ultrasonic vat (Cristófoli, Campo Mourão, PR, BR) with distilled water for 10 min ^26^.

After drying using absorbent paper, the specimens were etched with 37% phosphoric acid (Condac 37%, FGM, Joinville, SC, BR) for 10 min. The specimens were rinsed with distilled water, dried with air spray, and horizontally fixed in glass slides individuals. Each specimen was analyzed (1024X magnification) in a Laser confocal microscope (LEXT OLS4100; Olympus, Shinjuku-ku, Tokyo, Japan) with specific software (Olympus Stream; Olympus, Shinjuku-ku, Tokyo, JP), being obtained TIFF format images. The hybrid layer thickness in dentin was measured with Image J software as described by Morais et al. ^27^.

### Bond strength and failure mode analysis

60 specimens (10 mm length ×5 mm width) were obtained after the mesiodistal section of the 60 remaining teeth. The buccal surfaces of the specimens were polished (#180 silicon carbide sandpaper) in a polishing machine (DP-10; Panambra, Struers, Ballerup, DI) for dentin exposure and planning. After dentin exposure, specimens were polished using #320 and #600 sandpapers for 20 s each. Then, the specimens were placed into a polystyrene matrix mold (16.5 mm width x 25.0 mm length) embedded in acrylic resin (Classic Jet, São Paulo, SP, BR), and stored for 24 h.

After performing the bleaching, antioxidant, and restorative protocols, according to random allocation in the experimental groups (n=10), four composite resin cylinders were prepared on the buccal surface. A metallic matrix (1 mm high) containing four perforations with diameters equal to the resin cylinders (0.7 mm) was placed on the specimens, to delimit the area for the application of antioxidants and the performance of adhesive procedures. A transparent matrix (Tygon tube, R-3603, Saint-Gobain Performance Plastics, Maiami Lakes, FL, USA) with 0.7 mm internal diameter and 1.0 mm height was used to obtain the composite resin cylinders.

The micro-shear bond test was carried out after 24h storing in 99% relative humidity place at 37ºC. For the test, specimens were fixed in a metal matrix with the composite resin cylinders perpendicularly placed to a 500 KgF load cell. The composite cylinder base was held in position by an orthodontic wire (0.2 mm diameter) and all specimens were subjected to compressive loading (0.5 mm/min) with a mechanical testing machine (EMIC DL2000, São José dos Pinhais, PR, Brazil) until the displacing the composite resin specimens.

The bond strength values (in MPa) were obtained from the division maximum force (N) by the union area (mm^2^). An average value for each specimen was obtained from the four composite resin cylinders. After the bond strength test, the failed surfaces of each sample were analyzed with a stereomicroscope (SZ - PT; Olympus, Japan; 40X magnification). The failures were classified as adhesive (failure complete at the adhesive interface), cohesive (failure observed exclusively in dentin or resin), or mixed (when a combination of adhesive and cohesive failures was observed) ^26^.

### Data analysis

Firstly, descriptive analysis was performed, and the normality of data distribution was verified by the Shapiro-Wilk test (p=0.068 - fracture resistance; p=0.031 - hybrid layer; p=0.017 - bond strength). The fracture resistance values were analyzed by Analysis of Variance (ANOVA). Failure pattern differences were verified with the Qui-Square test. For hybrid layer thickness and bond strength data, Kruskal-Wallis and Dunn tests were used. All analyses were conducted with Statistical Package for Social Sciences software (SPSS for Windows, version 22.0, SPSS Inc. Chicago, IL, USA), with a 5% significance level.

## Results

### Fracture resistance and fracture pattern evaluation

The crown fracture strength values were significantly higher in the group without bleaching before restoration - REST (1508.96 ±148.15 N). However, the fracture strength did not differ significantly among bleached groups (p=0.065), regardless of antioxidant application (Table 2).

Favorable fractures were predominant in all groups (Table 3). No difference in the fracture pattern was observed between the evaluated groups (p=0.868).

**Table 2:** Dental crown fracture resistance (N) values after tooth bleaching with 38% hydrogen peroxide and antioxidants application in endodontically-treated teeth.

	REST	BL	SA	AT	CRAN	CAP
Mean	1508.96^a^	1077.70^a^	1166.72^a^	1104.51^a^	1110.91^a^	1143.08^a^
SD	148.15	233.39	118.17	157.12	109.01	162.69

^a,b^ Different letters shown statistical differences (p=0.065). REST: restoration without bleaching; BL - tooth bleaching without antioxidant; SA: 10% sodium ascorbate; AT: 10% alpha-tocopherol; CRAN: 5% cranberry; CAP: 0.0025% capsaicin.

**Table 3:** Failure pattern (absolute and relative frequencies) of the evaluated groups after fracture resistance test.

	REST	BL	SA	AT	CRAN	CAP
Favorable	8 (80.0)	7 (70.0)	9 (90.0)	8 (80.0)	7 (70.0)	7 (70.0)
Unfavorable	2 (20.0)	3 (30.0)	1 (10.0)	2 (20.0)	3 (30.0)	3 (30.0)

REST: restoration without bleaching; BL - tooth bleaching without antioxidant; SA: 10% sodium ascorbate; AT: 10% alpha-tocopherol; CRAN: 5% cranberry; CAP: 0.0025% capsaicin. No difference in fracture pattern was observed between evaluated groups (Qui-Square test, p=0.868)

### Hybrid layer evaluation

**Figure 1:**
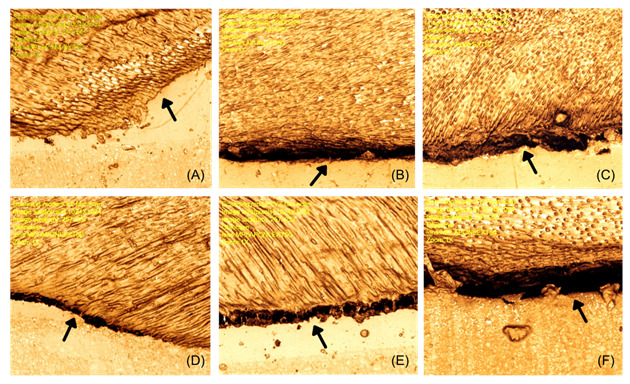
Representative images obtained by confocal laser microscopy (1024× magnification) showing hybrid layer formation analysis for all groups. A: REST - restoration without bleaching; B: BL - tooth bleaching; C: SA -10% sodium ascorbate; D: AT - 10% alpha tocopherol; E: CRAN - 5% cranberry; F: CAP - 0.0025% capsaicin.

The hybrid layer thickness in the group that was not subjected to bleaching was significantly higher than any other group (Table 4). Furthermore, the images (Figure 1) illustrate the hybrid layer thickness. In the REST group (Fig. 1, A) the hybrid layer is continuous (arrows). On other the hand, no difference was observed in all bleached groups, independent of antioxidant applications (Fig. 1, B-F).

**Table 4:** Hybrid layer thickness (in µm) values in dentin after bleaching and antioxidant protocols in dentin subjected to endodontic treatment.

	REST	BL	SA	AT	CRAN	CAP
Median	12.15^a^	0.15^b^	0.18^b^	0.15^b^	0.12^b^	0.19^b^
Min	5.36	0.00	0.00	0.00	0.00	0.00
Max	18.82	0.41	0.46	0.39	0.21	0.24

^a,b^ Different letters indicate significant statistical differences (p <0.05). REST: restoration without bleaching; BL - tooth bleaching without antioxidant; SA: 10% sodium ascorbate; AT: 10% alpha-tocopherol; CRAN: 5% cranberry; CAP: 0.0025% capsaicin.

### Bond strength and failure mode evaluation

The higher bond strength was observed in the non-bleached group - REST (14.48 MPa), which did differ significantly (p<0.001) from all bleached groups (range between 0.99 and 1.72 MPa). Moreover, no difference in the bond strength was observed in the groups treated with antioxidants (SA, AT, CRAN, CAP) compared with the bleached group with no antioxidant application (BL) (Table 5).

**Table 5:** Bond strength (MPa) to dentin after bleaching and antioxidant protocols in endodontically-treated teeth.

	REST	BL	SA	AT	CRAN	CAP
Median	14.48^a^	1.23^b^	1.32^b^	0.99^b^	1.01^b^	1.72^b^
Min	9.85	0.00	0.11	0.02	0.00	0.15
Max	23.82	3.28	3.33	3.01	3.01	3.38

^a,b^ Different letters indicate significant statistical differences (p <0.05). REST: restoration without bleaching; BL - tooth bleaching without antioxidant; SA: 10% sodium ascorbate; AT: 10% alpha-tocopherol; CRAN: 5% cranberry; CAP: 0.0025% capsaicin.

**Figure 2.**
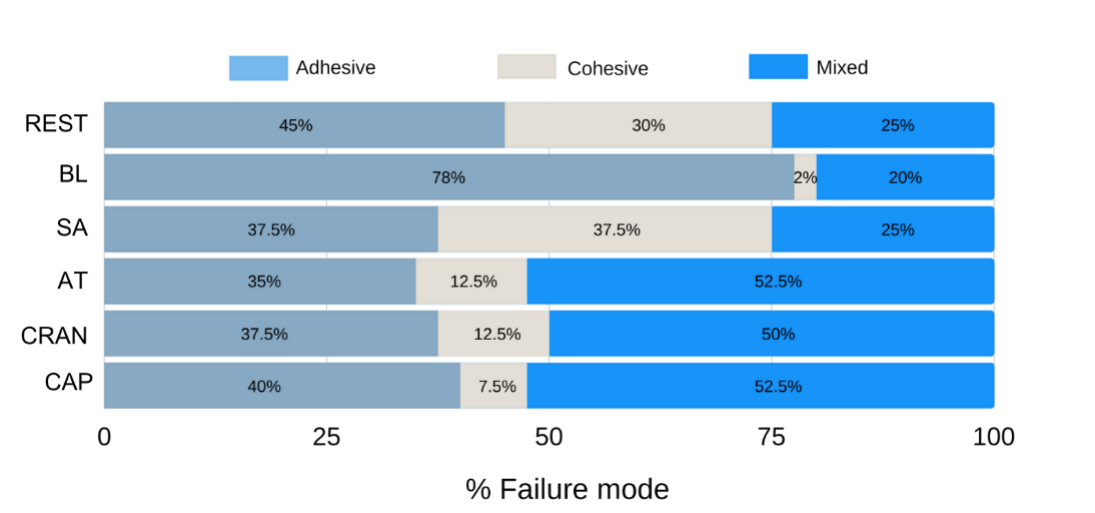
Failure mode analysis (%) for all groups after the micro-shear bond strength test.

The failure mode was significantly associated with the adopted protocol (p<0.001). The adhesive failure was the predominant mode in the groups without antioxidants application (REST and BL). The groups that received alpha-tocopherol, cranberry, or capsaicin application have mixed failures predominance. In addition, in the group with sodium ascorbate application, the adhesive and cohesive failures had similar occurrences (Figure 2).

REST - restoration without bleaching; BL - tooth bleaching; SA -10% sodium ascorbate; AT - 10% alpha tocopherol; CRAN - 5% cranberry; CAP - 0.0025% capsaicin.

Fisher’s exacttest (p-value <0.001).

## Discussion

This study suggests that solutions of sodium ascorbate, alpha-tocopherol, cranberry, and capsaicin did not neutralize free radicals resulting from tooth bleaching. Thus, these antioxidants did not show an effect on the dental fracture strength, hybrid layer formation, or bond strength of the adhesive to dentin bleached with 38% hydrogen peroxide.

Our findings show that a 10% sodium ascorbate solution does not affect dental fracture resistance. This finding is in contrast to Khoroushi et al. ^25^, who observed that fracture resistance increased in endodontically treated teeth after a combination of bleaching (35% in-office and 9.5% home-use) and sodium ascorbate application. However, it is relevant to emphasize that they used an antioxidant in a gel formulation, which is more effective than a solution formulation ^28^ and easier to apply. Besides, we used a higher hydrogen peroxide concentration (38%), causing more damage to the dental structure ^2^. However, there are reports in the literature that corroborate our findings on the non-effectiveness of sodium ascorbate on fracture resistance ^29^ ^,^ ^30^.

None of the other evaluated antioxidants promoted an increase in fracture resistance of endodontically treated teeth after bleaching. Leandrin et al. ^30^ observed a similar effect of alpha-tocopherol in solution after bleaching with 15% hydrogen peroxide. In addition, Jordão-Basso et al. ^29^ demonstrated that alpha-tocopherol gel also does not affect fracture resistance using a bleaching protocol and application time similar to the present study. Regarding the effects of cranberry and capsaicin solutions, the authors are not aware of any previous study that evaluated the possible increase in fracture resistance of the teeth after the application of these antioxidants. Thus, more studies are needed to verify whether these antioxidants can provide beneficial effects at other concentrations, formulations, and application times.

In our study, favorable fractures were observed in all groups. Thus, the tested antioxidants had no effect on the fracture pattern after the restoration of endodontically treated and bleached teeth. In contrast with our findings, bleaching protocol with 15% hydrogen peroxide with titanium dioxide particles and photoactivation with an LED system resulted in more non-favorable fractures compared to the same bleaching protocol with the application of sodium ascorbate or alpha-tocopherol ^30^. These different findings can be attributed to the variations in the bleaching protocols evaluated.

Restorative procedures after bleaching of endodontically treated teeth are a clinical challenge ^10^. Dentinal tubules are highly permeable to bleaching gels and act as reservoirs for oxygen-free radicals ^8^. A physiological mechanism for the release of these free radicals is through the pulp microcirculation ^31^. However, in endodontically treated teeth, this mechanism is not effective, causing free oxygen radicals to remain in the dentinal tubules for a longer time. Our findings demonstrated that the bleached tooth structure showed significantly reduced hybrid layer formation compared to the group that was not bleached. This finding is not surprising, as the literature reports that tooth bleaching can reduce resin penetrability into the tooth surface by approximately two-thirds ^32^.

In all groups that were subjected to bleaching, the effect on the formation of a hybrid layer was similar. Thus, there was no beneficial effect of applying any of the evaluated antioxidants. These results contrast with those of previous studies on the effect of sodium ascorbate on bleached enamel ^7^ ^,^ ^10^ ^,^ ^12^ ^,^ ^18^. However, it is recognized that restorative procedures are more challenging in dentin than in enamel. Previous studies demonstrated that higher concentrations of sodium ascorbate (35%) ^33^ and alpha-tocopherol (20%) ^34^ also did not effective in reversal bond strength to bleached dentin in non-vital teeth. In addition, the use of antioxidants in the solution form may not have represented an effective form of use. Thus, further studies on the effects of this antioxidant on bleached dentin are needed to confirm these findings.

Compared to other antioxidants, 6% cranberry has minimal effects on reversing the immediate bond strength of bleached enamel ^13^. At the best level of knowledge of the authors, there are no previous studies that have evaluated the effect of cranberry application after bleaching dentin. However, this limited effect on bleached enamel seems to justify that no improvement was observed with the application of this antioxidant in the adhesive performance to bleached dentin. Also are not aware of previous studies that have evaluated capsaicin solutions for this purpose. Our study did not demonstrate any beneficial effects of this antioxidant. Thus, our findings suggest that these antioxidants (cranberry and capsaicin) need to be further investigated, probably at other concentrations and application times, before recommendations for their use are made.

Unlike our findings, Sasaki et al. ^18^ observed a positive effect of the application of alpha-tocopherol in reversing the compromise of bleaching on the formation of a hybrid layer. These findings were related to whitening with carbamide peroxide. However, the impairment of adhesive procedures on a recently bleached surface is directly proportional to the concentration of the bleaching agent ^10^. This justifies that in the concentration and time of application evaluated in our study, alpha-tocopherol did not have a beneficial effect.

Although sodium ascorbate has shown satisfactory results in many studies, improving bond strength and avoiding adhesive failures ^8^ ^,^ ^18^ ^,^ ^35^, we did not observe these effects on the bond strength of dentin bleached with 38% hydrogen peroxide. Many studies with sodium ascorbate after bleaching with hydrogen peroxide (at concentrations ranging from 35%-40%) that showed positive effects on bond strength were performed in enamel ^36^ ^,^ ^37^, which is different from the present study, where bonding was carried out in dentin.

Although cranberry is a natural antioxidant containing OPC, and in vitro studies have reported that OPCs have 50 times more potent free radical neutralizing ability than sodium ascorbate and 20 times greater than vitamin E ^38^, 5% cranberry solution did not affect the adhesive interface and hybrid layer formation. Capsaicin also did not show good effects on bleached dentin. More studies are required to study these antioxidants, their concentrations, and formulations. Besides, this study demonstrated that cranberry has the potential to stain dentin. Thus, its application after bleaching may not be clinically viable.

The composite resin has a modulus of elasticity similar to dentin ^39^. This favors that teeth restored with composite resin have reduced stress concentration during load application ^40^. However, this benefit of composite resin filling is dependent on a good dentin/resin interface ^41^. Overall, this is the scientific basis to justify the similarity in fracture strength between the groups that received bleaching. This occurs since regardless of the application or type of antioxidant received the bond strength values are also no different from each other.

In this study, we chose to use bovine teeth. The ease to obtain the expressive number of teeth free of caries and necessary enamel defects was one of the reasons for this choice. In addition, greater uniformity is obtained in bovine teeth compared to human teeth. This improves the standardization of flat surface samples as required for some tests. There is evidence that bovine teeth can be a reliable substitute for human ones in bond strength studies ^42^ ^,^ ^43^. Although the extent of this applicability to fracture tests lacks evidence, we believe that the choice of analysis with bovine teeth does not compromise the findings of this study.

The findings of this study allow us to advance in the analysis of the effect of synthetic and natural clinically available antioxidants on restorative procedures in the dentin of endodontically treated teeth submitted to tooth bleaching. The importance of guiding clinical practice on the protocols to be adopted in these conditions requires that the feasibility of adopting this clinical step be better clarified. The antioxidants' effects on adhesive procedures in bleached enamel seem to be clearer ^7^ ^,^ ^10^, However, other antioxidants and different concentrations and/or times of application from those evaluated in this study need to be better investigated to favor adhesive restorative procedures also in bleached dentin.

## Conclusion

The different antioxidant solutions evaluated did not affect the fracture resistance of endodontically treated teeth. Favorable fractures were prevalent in all evaluated conditions, regardless of bleaching and the application of antioxidants. Moreover, the application of 10% sodium ascorbate, 10% alpha-tocopherol, 5% cranberry, or 0.0025% capsaicin solutions was not effective in terms of the hybrid layer thickness or bond strength about the bleached structure. However, the group restored without bleaching showed a significantly higher hybrid layer formation and bond strength than all other groups. In addition, in the groups that did not receive antioxidants, adhesive failures were predominant. Thus, our findings do not justify the adoption of this clinical step in the restorative protocol of endodontically treated and bleached teeth.

## References

[1] Frank AC, Kanzow P, Rödig T, Wiegand A (2022). Comparison of the Bleaching Efficacy of Different Agents Used for Internal Bleaching: A Systematic Review and Meta-Analysis. J Endod.

[2] Rodríguez-Martínez J, Valiente M, Sánchez-Martín MJ (2019). Tooth whitening: From the established treatments to novel approaches to prevent side effects. J Esthet Restor Dent.

[3] Kavitha M, Selvaraj S, Khetarpal A, Raj A, Pasupathy S, Shekar S (2016). Comparative evaluation of superoxide dismutase, alpha-tocopherol, and 10% sodium ascorbate on reversal of shear bond strength of bleached enamel: An in vitro study. Eur J Dent.

[4] de Oliveira DP, Teixeira EC, Ferraz CC, Teixeira FB (2007). Effect of intracoronal bleaching agents on dentin microhardness. J Endod.

[5] Sato C, Rodrigues FA, Garcia DM, Vidal CM, Pashley DH, Tjäderhane L (2013). Tooth bleaching increases dentinal protease activity. J Dent Res.

[6] Elfallah HM, Bertassoni LE, Charadram N, Rathsam C, Swain MV (2015). Effect of tooth bleaching agents on protein content and mechanical properties of dental enamel. Acta Biomater.

[7] Olmedo D, Kury M, Resende BA, Cavalli V (2021). Use of antioxidants to restore bond strength after tooth bleaching with peroxides. Eur J Oral Sci.

[8] Briso AL, Rahal V, Sundfeld RH, dos Santos PH, Alexandre RS (2014). Effect of sodium ascorbate on dentin bonding after two bleaching techniques. Oper Dent.

[9] Feiz A, Mosleh H, Nazeri R (2017). Evaluating the effect of antioxidant agents on shear bond strength of tooth-colored restorative materials after bleaching: A systematic review. J Mech Behav Biomed Mater.

[10] Rodríguez-Barragué J, Vola-Gelmini J, Skuras-Siedemburg M, Rivera-Gonzaga JA, Cuevas-Suarez CE (2021). Natural antioxidants to restore immediate bond strength to bleached enamel: Systematic review and meta-analysis of in vitro studies. J Esthet Restor Dent.

[11] Alqahtani MQ (2014). Tooth-bleaching procedures and their controversial effects: A literature review. Saudi Dent J.

[12] Elawsya ME, El-Shehawy TM, Zaghloul NM (2021). Influence of various antioxidants on micro-shear bond strength of resin composite to bleached enamel. J Esthet Restor Dent.

[13] Rahman H, Ansari MI, Khangwal M, Solanki R, Mansoori S (2021). Comparative evaluation of 6% cranberry, 10% green tea, 50% aloe vera and 10% sodium ascorbate on reversing the immediate bond strength of bleached enamel: In vitro study. J Oral Biol Craniofac Res.

[14] Freire A, Souza EM, de Menezes Caldas DB, Rosa EA, Bordin CF, de Carvalho RM (2009). Reaction kinetics of sodium ascorbate and dental bleaching gel. J Dent.

[15] Njus D, Kelley PM, Tu YJ, Schlegel HB (2020). Ascorbic acid: The chemistry underlying its antioxidant properties. Free Radic Biol Med.

[16] da Cunha LF, Furuse AY, Mondelli RF, Mondelli J (2010). Compromised bond strength after root dentin deproteinization reversed with ascorbic acid. J Endod.

[17] Garcia EJ, Oldoni TL, Alencar SM, Reis A, Loguercio AD, Grande RH (2012). Antioxidant activity by DPPH assay of potential solutions to be applied on bleached teeth. Braz Dent J.

[18] Sasaki RT, Flório FM, Basting RT (2009). Effect of 10% sodium ascorbate and 10% alpha-tocopherol in different formulations on the shear bond strength of enamel and dentin submitted to a home-use bleaching treatment. Oper Dent.

[19] Shi J, Yu J, Pohorly JE, Kakuda Y (2003). Polyphenolics in grape seeds-biochemistry and functionality. J Med Food.

[20] Luo XJ, Peng J, Li YJ (2011). Recent advances in the study on capsaicinoids and capsinoids. Eur J Pharmacol.

[21] Faul F, Erdfelder E, Lang AG, Buchner A (2007). G*Power 3: a flexible statistical power analysis program for the social, behavioral, and biomedical sciences. Behav Res Methods.

[22] Aranda-Garcia AJ, Kuga MC, Chavéz-Andrade GM, Kalatzis-Sousa NG, Hungaro Duarte MA, Faria G (2013). Effect of final irrigation protocols on microhardness and erosion of root canal dentin. Microsc Res Tech.

[23] Kuga MC, dos Santos Nunes Reis JM, Fabrício S, Bonetti-Filho I, de Campos EA, Faria G (2012). Fracture strength of incisor crowns after intracoronal bleaching with sodium percarbonate. Dent Traumatol.

[24] Pandolfo MT, Rover G, Bortoluzzi EA, Teixeira CDS, Rossetto HL, Fernandes PCDSV (2021). Fracture Resistance of Simulated Immature Teeth Reinforced with Different Mineral Aggregate-Based Materials. Braz Dent J..

[25] Khoroushi M, Feiz A, Khodamoradi R (2010). Fracture resistance of endodontically-treated teeth: effect of combination bleaching and an antioxidant. Oper Dent.

[26] Ramos ATPR, Garcia Belizário L, Venção AC, Fagundes Jordão-Basso KC, de Souza Rastelli AN, de Andrade MF (2018). Effects of Photodynamic Therapy on the Adhesive Interface of Fiber Posts Cementation Protocols. J Endod.

[27] Morais JMP, Victorino KR, Escalante-Otárola WG, Jordão-Basso KCF, Palma-Dibb RG, Kuga MC (2018). Effect of the calcium silicate-based sealer removal protocols and time-point of acid etching on the dentin adhesive interface. Microsc Res Tech.

[28] Breschi L, Cadenaro M, Antoniolli F, Visintini E, Toledano M, Di Lenarda R (2007). Extent of polymerization of dental bonding systems on bleached enamel. Am J Dent.

[29] Jordão-Basso KC, Kuga MC, Dantas AA, Tonetto MR, Lima SN, Bandéca MC (2016). Effects of alpha-tocopherol on fracture resistance after endodontic treatment, bleaching and restoration. Braz Oral Res.

[30] Leandrin TP, Alencar CM, Victorino KR, Dantas AA, Lima RO, Martins JC (2020). Is α-Tocopherol or Sodium Ascorbate Effective as Antioxidant on Fracture Resistance of Bleached Teeth?. J Contemp Dent Pract.

[31] Kodonas K, Gogos C, Tziafas D (2009). Effect of simulated pulpal microcirculation on intrapulpal temperature changes following application of heat on tooth surfaces. Int Endod J.

[32] Titley KC, Torneck CD, Smith DC, Chernecky R, Adibfar A (1991). Scanning electron microscopy observations on the penetration and structure of resin tags in bleached and unbleached bovine enamel. J Endod.

[33] Hansen JR, Frick KJ, Walker MP (2014). Effect of 35% sodium ascorbate treatment on microtensile bond strength after nonvital bleaching. J Endod.

[34] Harrison MS, Wang Y, Frick KJ, Moniz J, Walker MP (2019). Effects of Alpha-tocopherol Antioxidant on Dentin-composite Microtensile Bond Strength after Sodium Perborate Bleaching. J Endod.

[35] Briso AL, Toseto RM, Rahal V, dos Santos PH, Ambrosano GM (2012). Effect of sodium ascorbate on tag formation in bleached enamel. J Adhes Dent.

[36] Alencar MS, Bombonatti JF, Maenosono RM, Soares AF, Wang L, Mondelli RF (2016). Effect of Two Antioxidants Agents on Microtensile Bond Strength to Bleached Enamel. Braz Dent J.

[37] Boruziniat A, Manafi S, Cehreli ZC (2017). Synergistic effects of sodium ascorbate and acetone to restore compromised bond strength after enamel bleaching. Int J Esthet Dent.

[38] Subramonian R, Mathai V, Christaine Angelo JB, Ravi J (2015). Effect of three different antioxidants on the shear bond strength of composite resin to bleached enamel: An in vitro study. J Conserv Dent.

[39] Benetti AR, Peutzfeldt A, Lussi A, Flury S (2014). Resin composites: Modulus of elasticity and marginal quality. J Dent.

[40] Cabrera E, Macorra JC (2011). Microtensile bond strength distributions of three composite materials with different polymerization shrinkages bonded to dentin. J Adhes Dent.

[41] Santos AF, Meira JB, Tanaka CB, Xavier TA, Ballester RY, Lima RG (2010). Can fiber posts increase root stresses and reduce fracture?. J Dent Res.

[42] Soares FZ, Follak A, da Rosa LS, Montagner AF, Lenzi TL, Rocha RO (2016). Bovine tooth is a substitute for human tooth on bond strength studies: A systematic review and meta-analysis of in vitro studies. Dent Mater.

[43] de Carvalho MFF, Leijôto-Lannes ACN, Rodrigues MCN, Nogueira LC, Ferraz NKL, Moreira AN (2018). Viability of Bovine Teeth as a Substrate in Bond Strength Tests: A Systematic Review and Meta-analysis. J Adhes Dent.

